# Time trends and correlates of late presentation for HIV care in Northern Greece during the decade 2000 to 2010

**DOI:** 10.7448/IAS.15.2.17395

**Published:** 2012-10-11

**Authors:** Simeon Metallidis, Dimitrios Pilalas, Lemonia Skoura, Anna-Bettina Haidich, Olga Tsachouridou, Maria Papaioannou, Theofilos Chrysanthidis, Isidora Bakaimi, Zoe A Antoniadou, Apostolia Margariti, Nicolaos Malisiovas, Pavlos Nikolaidis

**Affiliations:** 1 Infectious Diseases Division, 1st Department of Internal Medicine, AHEPA University Hospital, Medical School, Aristotle University of Thessaloniki, Thessaloniki, Greece; 2 National AIDS Reference Centre of Northern Greece, Medical School, Aristotle University of Thessaloniki, Thessaloniki, Greece; 3 Department of Hygiene and Epidemiology, Medical School, Aristotle University of Thessaloniki, Thessaloniki, Greece

**Keywords:** HIV diagnosis, late presentation, risk factors, AIDS

## Abstract

**Background:**

The aim of our study was to assess the extent of late presentation for HIV care in Northern Greece during the period 2000 to 2010 and to explore correlations aiming to provide guidance for future interventions.

**Methods:**

HIV-positive patients with no prior history of HIV care at presentation and with a CD4 T cell count within three months from the first confirmatory Western blot result were eligible for this study. Late presentation and advanced HIV disease were defined in concordance with the recommendations of the European Late Presenter Consensus working group. Time trends in presentation status and risk factors linked to late presentation and advanced HIV disease were identified in multivariable logistic regression models. Additional analyses after multiple imputation of missing values were performed to assess the robustness of our findings.

**Results:**

The status at presentation was evaluated for 631 eligible HIV-positive individuals. Overall, 52.5% (95% CI: 48.6% to 56.4%) of patients presented late for HIV care and 31.2% (95% CI: 27.6% to 34.8%) presented with advanced HIV disease. Time trends were consistent with an improvement in the presentation status of our study population (*p<*0.001). Risk factors associated with late presentation in multivariable logistic regression were intravenous drug use, heterosexual HIV transmission, immigrant status and age at diagnosis.

**Conclusions:**

Despite the trend for improvement, a significant proportion of newly diagnosed HIV-positive patients present late for care. Targeted interventions with focus on social groups such as the elderly, persons who inject drugs, immigrants and individuals at risk for heterosexual HIV transmission are mandated.

## Background

Late presentation for HIV care has been recognized as a pressing public health issue which affects a substantial proportion of HIV-positive patients in the developed world, and it is associated with increased morbidity and mortality, increased healthcare expenses and possibly increased HIV transmission rates [1–3].

Reports from North America and Europe estimated late presentation to account for more than one-third of new HIV diagnoses [4–10]. Nevertheless, heterogeneity in the definition of late presentation limited the possibilities for direct comparisons and the identification of risk factors [11]. The HIV in Europe Initiative was launched in 2007 aiming to address barriers to early HIV diagnosis and to provide a framework for the study of late presentation [12].

According to data gathered from the national surveillance system, during the period January 2000 to October 2010, a total of 5360 individuals were diagnosed for the first time with HIV infection in Greece, and the number of new HIV diagnoses during the last three years of the study period was estimated to be approximately 50 new cases per million person-years [13].

The main objective of our retrospective study was to examine patterns of presentation for HIV care in Northern Greece and to identify risk factors associated with late presentation.

## Methods

### Patients

Patients eligible for this study received their first confirmatory Western blot result during the period from January 2000 to October 2010 and presented for HIV care in the Infectious Diseases Division, AHEPA University Hospital in Thessaloniki, Greece. AHEPA University Hospital is the single centre to provide HIV care services in Central Macedonia (approximately 2 million inhabitants according to data from the 2011 census) and the wider region of Northern Greece. Adjacent centres providing HIV care services are located in Alexandroupolis (Thrace) and Athens at a distance of 300 and 500 km, respectively. Eligibility criteria also included no previous history of HIV care and a CD4 cell count within three months since HIV diagnosis.

Data on sex, patient age at diagnosis, calendar year of HIV diagnosis, risk factors for acquisition of HIV disease, patient origin, CD4 T cell count at diagnosis, baseline viral load and AIDS-defining events at presentation were extracted from the database. Data were recorded in the database retrospectively until 2008 with review of medical files and prospectively thereafter. All patients provided informed consent to maintain data for therapeutic and research purposes and handling of the data ensured patient anonymity. The study received approval from the Bioethics Committee of the Medical School.

### Definitions

A consensus definition developed under the auspices of the HIV in Europe Initiative was implemented in order to describe patterns of presentation for HIV care [14].

Late presentation: CD4 cell count below 350 cells/µL or AIDS-defining event at presentation.

Patients with advanced HIV disease: CD4 cell count below 200 cells/µL or AIDS-defining event at presentation.

In order to ensure that the status at presentation was actually captured, CD4 cell count and AIDS-defining event diagnosis had to be reported within three months since the date of the confirmatory Western blot result. In case a confirmatory CD4 cell count was obtained within a month after the first CD4 cell count measurement, the mean of the two measurements was used for purposes of analysis.

### Statistical methods

We evaluated temporal trends with the Cochran-Armitage trend test. Continuous variables were assessed with the Kruskal-Wallis test since they were not normally distributed.

Logistic regression models were employed in order to assess predictors of late presentation or advanced HIV disease. Predictors that were significantly (*p<*0.05) associated with late presentation or advanced HIV infection in univariate analyses were introduced in a multivariable model by forced entry. The *c* statistic (area under receiver operating characteristic curve) was used to assess the discriminative performance of the logistic regression models and the Hosmer-Lemeshow test of goodness of fit was used to evaluate the fit of the model. In addition, we conducted an exploratory analysis introducing a piecewise linear term for calendar year.

In our primary analysis, unknown risk factor for HIV infection was used as a predictor in order to evaluate risk associated with patient unwillingness to disclose sensitive personal information. The robustness of our findings was also tested after multiple imputation was performed under the assumption of missing at random pattern of missingness [15]. Although sensitive personal information suggests the possibility of a not-at-random pattern of data missingness, existing data do not permit the estimation of the true distribution of risk factors in the population. Thus, multiple imputation was deemed as most appropriate to address missing data and to evaluate the robustness of our findings.

Missing values were observed in 26% of patients. In 21.6% of cases, a risk factor for HIV infection could not be established at first clinic visit or was missing. Viral load at baseline was missing in 5.9% of cases. Multiple imputation was performed to generate 20 complete datasets based on sex, year of HIV diagnosis, patient age, status at presentation, country of origin, viral load and risk factor for HIV infection as implemented in SPSS Multiple Imputation command. Statistical analyses were conducted with SPSS Statistics 17 (SPSS Inc. Chicago, IL, USA).

## Results

### Study population characteristics

A total of 631 patients met the eligibility criteria. No specific time pattern regarding the proportion of individuals excluded due to the requirement for a CD4 cell count within three months since diagnosis was observed. The study population predominantly comprised of male patients (83.5%, 527/631). The most prevalent risk factor for HIV infection was men having sex with men (MSM; 53.1%, 335/631). Heterosexual contact as a risk factor was reported in 20.6% of cases (130/631), and intravenous drug use was reported in 4.8% of cases (30/631). A very small proportion of patients reported more than one risk factors for HIV infection (less than 1%). For purposes of analysis, the risk factor perceived from patient history as more relevant for the acquisition of HIV infection was used. Three patients reported multiple transfusions as a risk factor, and due to the limited number of patients in this group, they were excluded from logistic regression models. Notably, in 21.1% of cases no risk factor for HIV infection was reported (133/631). Ten percent of patients (63/631) were not of Greek origin. Mean age at diagnosis was 37.4 years (standard deviation, SD: 12.4). Finally, median CD4 cell count at diagnosis was 341 cells/μL (interquartile range, IQR: 160 to 524), and mean plasma HIV RNA was 4.78 log cop/mL (SD: 0.98).

Time trends in the synthesis of the study population suggested an increase in the proportion of male patients (*p=*0.001). Consistently, MSM as a risk factor for HIV acquisition became more prevalent over time (*p<*0.001), whereas the heterosexual contact became less prevalent. The frequency of intravenous drug use was not significantly altered over the study period (*p=*0.469). A trend for decrease in the percentage of patients with no reported risk factors for HIV infection was also observed (*p=*0.042). The proportion of patients of foreign origin remained stable over time (*p=*0.406). A trend for increase in age at presentation was observed over time (*p=*0.088).

### Late presentation and advanced HIV disease

Overall, 52.5% (331/631, 95% confidence intervals, CI: 48.6% to 56.4%) of patients presenting for HIV care during the period 2000 to 2010 were classified as late presenters according to the consensus definition. The proportion of patients who presented with advanced HIV disease was 31.2% (197/631, 95% CI: 27.6% to 34.8%).

Male patients presented late for HIV care in 51% of cases (269/527) vs. 59.6% (62/104) for female patients (*p=*0.11). Advanced HIV disease also affected male and female patients in similar proportions (31.3% and 30.8%, respectively).

**Figure 1:**
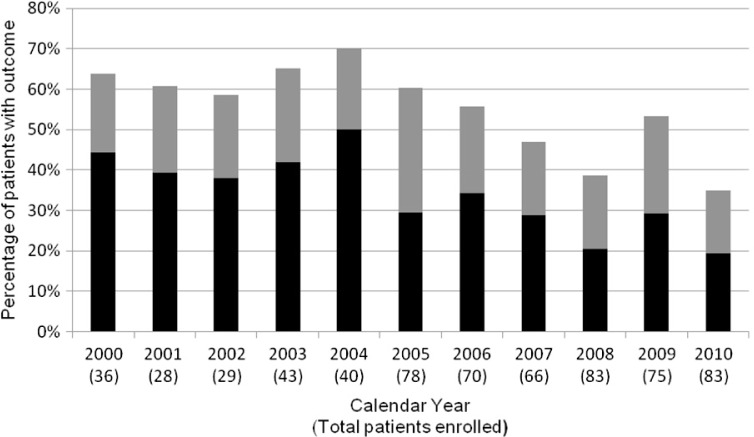
Presentation for HIV care in Northern Greece. The black component of each bar represents patients with advanced HIV disease. Each bar as a whole represents the proportion of late presenters. The numbers in parentheses represent the total number of eligible patients for the corresponding calendar year.

Temporal trends in presentation for HIV care revealed a decrease in the proportion of both late presenters and patients with advanced HIV disease (*p<*0.001 in both cases) (Figure 1). A sensitivity analysis of the evolution of CD4 T cell count at presentation during the study period corroborated these findings (*p<*0.001). Sensitivity analyses assessing separately trends in late presentation during the periods of retrospective and prospective data collection produced consistent results (*p<*0.001 and *p=*0.02, respectively).

### Factors associated with patient status at presentation

Factors associated with late presentation and advanced HIV disease in multivariable logistic analysis are presented in Table 1. The *c* statistic was 0.662 (95% CI: 0.620 to 0.705) for the late presentation logistic regression model and 0.683 (95% CI: 0.639 to 0.726) for the advanced HIV logistic regression model. The fit of both models was good as indicated by the Hosmer-Lemeshow goodness of fit test (*p=*0.60 and *p=*0.17, respectively). The introduction of a piecewise linear term for calendar year (knot in year 2004) was not supported in multivariable models for late presentation and advanced HIV disease.

**Table 1:** Multivariable logistic regression models for late presentation and advanced HIV disease

	Late presenters		Late presenters (multiple imputation)		Patients with advanced HIV disease		Patients with advanced HIV disease (multiple imputation)	
				
Predictor	Odds ratio (95% CI)	*p*	Odds ratio (95% CI)	*p*	Odds ratio (95% CI)	*p*	Odds ratio (95% CI)	*p*
Calendar year of diagnosis (per one year increase)	0.90 (0.85 to 0.95)	<0.001	0.90 (0.85 to 0.95)	<0.001	0.90 (0.84 to 0.95)	<0.001	0.90 (0.84 to 0.95)	<0.001
Age at diagnosis (per 10 years increase)	1.34 (1.16 to 1.55)	<0.001	1.34 (1.17 to 1.55)	<0.001	1.47 (1.26 to 1.69)	<0.001	1.47 (1.28 to 1.69)	<0.001
Risk factor for HIV infection (reference MSM)								
Heterosexual contract	1.48 (0.95 to 2.30)	0.086	1.62 (1.06 to 2.47)	0.027	1.37 (0.87 to 2.16)	0.174	1.31 (0.83 to 2.07)	0.246
Intravenous drug use	2.19 (0.98 to 4.90)	0.057	2.31 (1.02 to 5.25)	0.046	2.23 (1.02 to 4.86)	0.044	2.19 (1.03 to 4.63)	0.041
Unknown	1.26 (0.83 to 1.92)	0.281			1.32 (0.84 to 2.06)	0.231		
Origin other than Greek	1.65 (0.93 to 2.93)	0.088	1.52 (0.84 to 2.73)	0.166				

MSM, men having sex with men.

Additional analyses were performed on datasets where unknown risk factor for HIV infection was multiply imputed, and the results were similar (Table 1).

## Discussion

We undertook a retrospective single centre study of the epidemiology of late presentation for HIV care during the period 2000 to 2010 in Northern Greece. We reported data on a sample of 631 patients who presented for the first time for HIV care in the HIV clinic of the AHEPA University Hospital. This patient sample represents 11.8% (631/5360) of the patients diagnosed for the first time with HIV infection in Greece during the study period, according to mandatory reportable data from the Greek national HIV/AIDS surveillance system [13].

The HIV epidemic in Northern Greece is predominated by subtype B HIV-1 infection with gradual increase in the prevalence of subtype A in the native population and during the study period parallels the HIV epidemic as documented in previous studies conducted in Athens [16,17]. Furthermore, the synthesis of the HIV-positive population in our region is comparable to the demographic characteristics of the HIV-positive population in Greece as reported up to October 2010 in the national HIV/AIDS surveillance system [13].

The consensus definition of the European Late Presenter Consensus Group was adopted for analysis [14]. According to this definition, patients who present with less than 350 CD4 cells/mm^3^ or an AIDS-defining event and, thus, fail to derive maximal benefit from antiretroviral therapy are classified as *late presenters*. Those who present with less than 200 CD4 cells/mm^3^ or an AIDS-defining event are classified as patients with *advanced HIV disease* and are at increased risk of death [18]. Wide implementation of the consensus definition will address the previously observed heterogeneity in the definition of late presentation and will provide a framework to evaluate the effectiveness of measures and policies to address late presentation.

In our study population, 52.5% (331/631, 95% CI: 48.6% to 56.4%) of patients presenting for HIV care during the period 2000 to 2010 were classified as late presenters. The proportion of patients who presented with advanced HIV disease was 31.2% (197/631, 95% CI: 27.6% to 34.8%). The estimated prevalence is consistent with results from previous studies in the United States of America and Europe [4,7–9]. Time trends provide evidence that status at presentation has improved during the past decade (*p<*0.001). However, a substantial proportion of patients remain undiagnosed until late in the course of disease.

Risk factors associated with late presentation and advanced HIV disease were also consistent with results from previous studies and were robust to the influence of missing data [11]. Adjusting for other factors, older age was strongly associated with late presentation (*p<*0.001). Heterosexual contact and intravenous drug use, compared to MSM, conferred increased risk by a factor of 48% and 19%, respectively. Immigrant status conferred increased risk by a factor of 65%. Although the HIV epidemic in Greece predominantly affects MSM, populations such as persons who inject drugs and immigrants are at higher risk for late presentation possibly due to barriers in healthcare [13,14]. Prevention and early detection programmes should extend to the entire spectrum of the sexually active population in order to tackle the high prevalence of late presentation in the elderly.

A considerable proportion of patients did not report a known risk factor for acquisition of HIV disease. We hypothesized that failure to report a risk factor for acquisition of HIV disease may be a result of social stigma, and we investigated the possibility that it would be associated with delayed linkage to care. However, our findings do not provide evidence in favour of this hypothesis (*p=*0.281).

Of concern, the discriminative performance of the multivariable logistic regression models for late presentation and advanced HIV disease was poor (*c* statistic<0.7). The *c* statistic serves to assess how well the model discriminates cases from non-cases, and in order to achieve a substantial improvement in its value, combinations of new risk factors should be evaluated [19].

Limitations of our study include the retrospective data collection and patient reluctance to report risk factors for acquisition of HIV infection due to related stigma. Furthermore, our data precluded us from evaluating other predictors which may be relevant for status at presentation such as social status and education. Finally, the generalizability of the findings of our study may be limited since the study sample came from a single centre.

To our knowledge, our study is the first to report rates and correlates of late presentation in Greece. Rates of late presentation seem to be declining. These findings, albeit optimistic, cannot provide reassurance for the future. The financial crisis and the austerity measures imposed drive more people to the margin and may substantially reduce state expenditure on public health. Constant vigilance is required to face emerging challenges with regard to the prevention, diagnosis and treatment of HIV infection.

## Abbreviations

CI, confidence intervals; IQR, interquartile range; MSM, men having sex with men; SD, standard deviation.
